# Is Angiostrongylosis a Realistic Threat for Domestic Cats?

**DOI:** 10.3389/fvets.2020.00195

**Published:** 2020-04-15

**Authors:** Angela Di Cesare, Simone Morelli, Mariasole Colombo, Giulia Simonato, Fabrizia Veronesi, Federica Marcer, Anastasia Diakou, Roberto D'Angelosante, Nikola Pantchev, Evanthia Psaralexi, Donato Traversa

**Affiliations:** ^1^Faculty of Veterinary Medicine, University of Teramo, Teramo, Italy; ^2^Department of Animal Medicine, Production and Health, University of Padua, Padua, Italy; ^3^Department of Veterinary Medicine, University of Perugia, Perugia, Italy; ^4^Faculty of Veterinary Medicine, Aristotle University of Thessaloniki, Thessaloniki, Greece; ^5^IDEXX Laboratories, Ludwigsburg, Germany

**Keywords:** *Angiostrongylus chabaudi*, wildcat, cat, angiostrongylosis, angio detect™

## Abstract

Three species of *Angiostrongylus* have been found in felids thus far, i.e., *Angiostrongylus chabaudi, Angiostrongylus felineus* and *Angiostrongylus vasorum*. *Angiostrongylus chabaudi* lives in the right heart and pulmonary arteries of the definitive natural host, the European wildcat (*Felis silvestris*), and non-patent infections have been reported in domestic cats (*Felis catus*). *Angiostrongylus felineus*, described in the Puma yaguarondi (*Herpailurus yagouaroundi*), has never been reported in domestic felids, while recently a non-patent infection by *A. vasorum* was unequivocally described in a *F. catus*. Nonetheless, epizootiological and clinical relevance of angiostrongylosis in domestic cats are practically unknown. This study investigated whether cases of angiostrongylosis may be missed in cats living in areas enzootic for *Angiostrongylus* spp. and other metastrongyloids. Overall, 100 cats that were either positive (n.50) or negative (n.50) for metastrongyloid larvae at the Baermann's test, were examined for *Angiostrongylus* spp. with DNA-based methods and with the serological test Angio Detect™ for circulating antigen. The PCR analysis confirmed the copromicroscopy results, where 25 cats scored positive for *Aelurostrongylus abstrusus*, 16 for *Troglostrongylus brevior* and 9 for both, while no cats were positive for *Angiostrongylus*-like larvae, including *A. chabaudi*. None of the 100 sera samples scored positive at the Angio Detect™ test. These data suggest that currently feline angiostrongylosis is a minor parasitosis for domestic cats. Nevertheless, it cannot be excluded that the epizootiological drivers which have favored the spillover of *A. vasorum* and *T. brevior* from wildlife to dogs and cats, could promote the emergence of feline angiostrongylosis, with an unpredictable health impact.

## Introduction

Canine and feline cardio-pulmonary nematodes are emerging throughout Europe due to different factors (1, 2). A role in the spreading of some extra-intestinal parasites can be played by bridging infections between wildlife and domestic hosts (3, 4). This has recently led to the establishment in domestic animals of parasites which were previously considered to be affiliated only to their definitive wild hosts. As key examples, among these “new” parasites, *Angiostrongylus vasorum* causing canine angiostrongylosis, *Troglostrongylus brevior* causing feline troglostrongylosis and *Oslerus rostratus* causing feline oslerosis were previously considered typical of red foxes (*Vulpes vulpes*), European wildcats (*Felis silvestris*) and the lynx (*Lynx* spp.), respectively. These nematodes have now a major impact on animal health, causing potentially fatal diseases in domestic canine and feline populations (4–6).

While angiostrongylosis in dogs has become a well-known disease in Europe, our knowledge on the infections caused by *Angiostrongylus* spp. in felines is very poor. *Angiostrongylus chabaudi* is a metastrongyloid nematode that was first described in wildcats from Central Italy in 1957 (7), and that has not been reported again until few years ago, when it was found in a domestic cat (*Felis catus*) from Sardinia (Italy) as a non-patent infection (8). Then, this parasite has been documented in another non-patently infected domestic cat from Central Italy (9) and in wildcats from Germany (10), Italy (11), Greece (12, 13), Romania (14), Bulgaria (15), Bosnia and Herzegovina (16). Recently, the European wildcat has been shown to be the definitive host of *A. chabaudi*, with a demonstration of patent infections and presence of first stage larvae (L1) in the feces for the first time (12). The clinical importance of angiostrongylosis caused by *A. chabaudi* in domestic cats is virtually unknown, although pathological lesions (e.g., granulomatous pneumonia, parenchymal hemorrhagies, alveolar emphysema, subendothelial proliferation and oedema, thrombosis and hyperplasia/hypertrophy of the pulmonary arteries) have been reported in both wild and domestic cats (9, 11, 12, 16). With regard to other *Angiostrongylus* species, the ability of *A. vasorum* to infect felid hosts was shown under experimental conditions (17, 18), and recently, a case of natural infection has been described in a domestic cat from Switzerland (19). However, to date patent infections caused by *A. vasorum* in *F. catus* have never been described neither in experimental nor in natural conditions. Few years ago, *Angiostrongylus felineus* has been described for the first time in the Puma yaguarondi (*Herpailurus yagouaroundi*) from Brazil (20), but at present no cases of infection have been documented in domestic cats.

The diagnosis of feline cardio-respiratory parasitic infections is currently based on the morphometric and morphologic identification of L1 shed in faces using the Baermann test (21). A key limitation in diagnosing possible angiostrongylosis in domestic cats is due to the fact that at the moment there is no any demonstrations of patent infections. This absence of data could have been caused by missed diagnosis due to a certain similarity of microscopic features of *Angiostrongylus* with those of other metastrongyloids more commonly detected in fecal samples of cats (i.e., *Aelurostrongylus abstrusus* and *T. brevior*) (12).

DNA-based assays are powerful to identify metastrongyloid L1s in the feces of cats (22), thus would have the potential to reveal missed diagnosis at the microscopic analysis. The rapid serological test Angio Detect™ (IDEXX Laboratories Inc.) is able to detect circulating antigens produced by *A. vasorum* infecting dogs (23). Interestingly, this test has recently proved useful for the detection of other *Angiostrongylus* species, i.e., *A. chabaudi* in European wildcats and *Angiostrongylus daskalovi* in badgers (24). Being able to detect *Angiostrongylus* spp. circulating antigens, this test could also have a potential to diagnose feline angiostrongylosis.

The present study has aimed at investigating for the first time whether angiostrongylosis is a potentially missed, neglected or underestimated disease in domestic cats by using DNA-based and serological tests.

## Materials and Methods

One-hundred domestic cats from regions of Italy and Greece enzootic for *Angiostrongylus* spp. and major feline cardio-respiratory nematodes (2, 9, 11–13) were examined for the presence of metastrongyloid larvae. Animals were selected as a convenient dataset, as they were referred for routine clinical examinations and/or presence of compatible clinical signs. All animals were not sampled purposely for the present study and were not subjected to unnecessary suffering. Only the surplus material deriving from blood samplings expressly requested by the owner and/or indicated by the examining veterinarian for other diagnostic purposes was used in this study. Healthy animals were sampled providing that they were from enzootic areas and had a free-roaming lifestyle. From one to three consecutive fecal samples were examined for each cat by a Baermann's examination (2). Cats were divided in two groups, i.e., cats that scored either positive (G1) or negative (G2) for metastrongyloid larvae at the fecal test. All larvae collected at the Baermann test were examined using morphological and morphometric keys (12, 21). All Baermann's sediments were subjected to a multiplex-PCR as previously described (22) that is able to simultaneously detect *A. chabaudi, Ae. abstrusus*, and *T. brevior*. An individual blood sample was obtained for each cat with the owner's consent. Samples were collected for veterinary hematological examinations (e.g., routine or other diagnostic tests). Samples were subjected to centrifugation to obtain the serum. All sera samples were examined using the Angio Detect™ test (IDEXX Laboratories, Westbrook, Maine, USA), according to the manufacturer's instructions. More details are reported in Schnyder et al. (23).

## Results and Discussion

Out of the 50 Baermann-positive cats (G1), 25 were microscopically positive for *Ae. abstrusus*, 16 for *T. brevior* and nine were positive for both nematodes. None of the samples scored positive for *Angiostrongylus*-like larvae nor for *O. rostratus* (the latter was not surprising as *O. rostratus* larvae usually do not migrate in water). PCR results confirmed the microscopic identification of the L1s retrieved in the Baermann sediment and there were no PCR-positive for samples negative at the copromicroscopy. All sera samples scored negative for circulating *Angiostrongylus* antigen at the Angio Detect™ (Table 1).

**Table 1:** Results of the Baermann examination confirmed by molecular analysis (*) and of the serological Angio Detect^TM^ test performed in the present study.

	**Italy n/tot (%)**	**Greece n/tot (%)**	**Total n/tot (%)**
Aelurostrongylus abstrusus^*^	23/64 (35.9)	2/36 (5.5)	25/100 (25)
Troglostrongylus brevior^*^	15/64 (23.4)	1/36 (2.7)	16/100 (16)
*Aelurostrongylus abstrusus* + Troglostrongylus brevior^*^	8/64 (12.5)	1/36 (2.7)	9/100 (9)
Angiostrongylus chabaudi^*^	0/64 (0)	0/36 (0)	0/100 (0)
Angio Detect^TM^	0/64 (0)	0/64 (0)	0/100 (0)

Although preliminary, this study suggests that cats living in areas where *Angiostrongylus* is present even with high infection rates (2, 11–13), are realistically at a null or minor risk of acquiring angiostrongylosis. The absence of *A. chabaudi* L1s in potentially infected cats could be due to the inability of this metastrongyloid to reach the adult stage in domestic hosts (8, 9). To date, indeed, only two cases of *A. chabaudi* infection have been described in domestic cats, where necropsy examinations showed immature nematodes in the pulmonary arteries without evidence of L1 in feces (8, 9). In a recent survey, the Angio Detect™ test showed a 97.1% sensitivity and a 98.9% specificity in detecting *A. vasorum* infection in dogs (25) and another study carried out on wildcats and badgers positive for *A. chabaudi* and *A. daskalovi*, respectively, showed 100% correlation between necropsy results, confirmed by molecular assays, and serologic positivity to the Angio Detect™ test (24). The sero-negativity of G1 cats suggests a lack of cross-reactivity between the different lungworm species infecting cats. In fact, it seems that the Angio Detect™ test can cross-react exclusively between species of the genus *Angiostrongylus* (24). This might be also explained by their colonization of blood vessels and thus the direct availability of circulating antigen in blood samples, as it is the case with the heartworm, *Dirofilaria immitis*. It can be assumed that this rapid assay has an elevated sensitivity, although not 100% (25), either in wildcats or in domestic cats, because its reliability does not seem to be influenced by the tested species, having been used successfully in non-canine hosts (24). Furthermore, the Angio Detect™ is able to detect the infection also in absence of *Angiostrongylus* spp. L1 in feces, as long as the parasites have reached adulthood (25). Therefore, it is unlikely that the cats of the present study were false negative, having been tested with three different diagnostic methods and the serologic negativity is most probably due to a real absence of *A. chabaudi, A. vasorum*, or other *Angiostrongylus* species. In fact, cats of G1, shedding larvae, would have scored positive at least at one of the tests performed in the present study. This is supported by the fact that all cats of both G1 and G2 scored negative for *Angiostrongylus* spp., other than the Angio Detect^TM^ test, also at the molecular analysis for *A. chabaudi*, which seems to be the major *Angiostrongylus* infecting felines. Nonetheless, it should be considered that the antigens detectable with this rapid assay are produced by adult *Angiostrongylus* worms (23, 26) and that *A. chabaudi* and *A. vasorum* are most probably unable to reach adulthood producing detectable antigens in domestic cats (8, 9, 19). Indeed, a nematode found in a pulmonary artery of a domestic cat, whose section size and content were suggestive of an adult stage, was identified as *A. vasorum* at PCR analysis despite the cat scored negative at the Angio Detect™ test (19). Such negativity could be due to different reasons, including a <100% sensitivity of the test (25) or to the fact that the first antigens are detectable 9 weeks post-infection (23). As development of *A. vasorum* to adult may occur as early as 28 days post-infection (27) false negative results might occur with this test even in presence of adult nematodes. The herein examined cats could have scored as false negatives only in a similar scenario, where *A. vasorum* infection confirmation could be possible only with necropsy and molecular analysis on the nematodes retrieved in the arteries. Although this possibility cannot be ultimately excluded, these results indicate that angiostrongylosis, at present, is still an infection of minor importance for domestic cats. However, possible bridging infections by *Angiostrongylus* in future, especially *A. chabaudi*, between wildcats and domestic cats cannot be ruled out. In fact, different factors are influencing domestic and wild fauna interactions, e.g., urbanization and reduction of wildlife habitats (2). Thus, spill-over events similar to those that have likely occurred for *A. vasorum* in dogs and *T. brevior* in cats (4, 6) may indicate that similar eco-epizootiological modifications could have the potential to influence biology and epizootiology of related parasites belonging to the genus *Angiostrongylus*. A possible spreading of feline angiostrongylosis could have a severe clinical relevance in domestic cats, as happened for canine angiostrongylosis. Angiostrongylosis by *A. vasorum* in foxes, i.e., the natural reservoir, appears to be milder that in dogs, with a moderate impact on the general health status, and with pathological lesions suggestive of a mainly chronic course (28–30), while canine angiostrongylosis is a well-known disease with a possible fatal outcome (6).

In conclusion, domestic cats seem to be at low risk of angiostrongylosis, even in areas where wildcats and domestic cats live in sympatry and where different species of *Angiostrongylus* occur.

It is interesting to note also that the worldwide spread land snail *Cornu aspersum* may act as intermediate host of *A. chabaudi, A. vasorum*, and *Ae. abstrusus* (31–33). Despite *A. vasorum* and *Ae. abstrusus* may be present in the same geographic areas, possibly developing in *C. aspersum*, this is not the case of *A. chabaudi*. This could be due to the very low presence of this nematode in its reservoirs, that is also reflected by a low presence of infective stages in mollusks and paratenic hosts.

As future outbreaks of feline angiostrongylosis with an unforeseeable impact on feline health should be taken into account, constant epizootiological surveillance appears to be crucial. The use of Angio Detect^TM^ is promising for its use in feline medicine under both clinical and epizootiological standpoints although the diagnostic efficiency of this test in cats that have non-patent infections remains to be understood. As the identification of *Angiostrongylus* L1 in cats can be impaired by the similarity in morphologic and morphometric features with other metastrongyloids that can be usually found at the Baermann's examination (12, 21), the use of combined diagnostic approaches including copromicroscopy, DNA-based assays and rapid serological kits may minimize the chances of false negative results and is herein encouraged in cats that could be at risk of infection by *Angiostrongylus*.

## Data Availability Statement

The datasets generated for this study are available on request to the corresponding author.

## Ethics Statement

Ethical approval for this study was not required according to national/local legislation because no cats were sampled or subjected to unnecessary suffering. The animals included were not bled for this study as only the surplus material deriving from blood samplings expressly requested by the owner and/or indicated by the examining veterinarian for other diagnostic purposes was used in this study.

## Author Contributions

ADC, SM, and DT participated in study activities and in drafting and revising the manuscript. MC, GS, FV, FM, AD, RD'A, and EP participated in the field and laboratory work. NP participated in the study design and in interpreting the serological results. All authors have participated in critically revising the manuscript.

### Conflict of Interest

NP was employed by the company IDEXX Laboratories, Inc. The remaining authors declare that the research was conducted in the absence of any commercial or financial relationships that could be construed as a potential conflict of interest.
